# Contrast-enhanced optical coherence tomography with picomolar sensitivity for functional *in vivo* imaging

**DOI:** 10.1038/srep23337

**Published:** 2016-03-18

**Authors:** Orly Liba, Elliott D. SoRelle, Debasish Sen, Adam de la Zerda

**Affiliations:** 1 Molecular Imaging Program at Stanford, Stanford University, 299 Campus Drive, Stanford, California 94305, USA; 2 Bio-X Program, Stanford University, 299 Campus Drive, Stanford, California 94305, USA; 3 Biophysics Program, Stanford University, 299 Campus Drive, Stanford, California 94305, USA; 4 Departments of Structural Biology, Stanford University, 299 Campus Drive, Stanford, California 94305, USA; 5 Electrical Engineering, Stanford University, 299 Campus Drive, Stanford, California 94305, USA

## Abstract

Optical Coherence Tomography (OCT) enables real-time imaging of living tissues at cell-scale resolution over millimeters in three dimensions. Despite these advantages, functional biological studies with OCT have been limited by a lack of exogenous contrast agents that can be distinguished from tissue. Here we report an approach to functional OCT imaging that implements custom algorithms to spectrally identify unique contrast agents: large gold nanorods (LGNRs). LGNRs exhibit 110-fold greater spectral signal per particle than conventional GNRs, which enables detection of individual LGNRs in water and concentrations as low as 250 pM in the circulation of living mice. This translates to ~40 particles per imaging voxel *in vivo*. Unlike previous implementations of OCT spectral detection, the methods described herein adaptively compensate for depth and processing artifacts on a per sample basis. Collectively, these methods enable high-quality noninvasive contrast-enhanced imaging of OCT in living subjects, including detection of tumor microvasculature at twice the depth achievable with conventional OCT. Additionally, multiplexed detection of spectrally-distinct LGNRs was demonstrated to observe discrete patterns of lymphatic drainage and identify individual lymphangions and lymphatic valve functional states. These capabilities provide a powerful platform for **mo**lecular imaging and characteri**z**ation of tissue noninvasively **a**t cellular **r**esolu**t**ion, called MOZART.

Recently, major advances have been made in the field of *in vivo* functional imaging. Modalities with high depth of penetration in tissue such as positron emission tomography and photoacoustic imaging have become powerful techniques through the use of molecular contrast agents^1^,^2^,^3^. However these techniques suffer from poor spatial resolution, and consequently a single image voxel may contain between 10^3^ to 10^7^ cells^4^,^5^,^6^. Other modalities such as optical microscopy easily achieve sub-cellular spatial resolution but lack the ability to image more than several hundred microns deep in turbid tissue^7^,^8^. Given current limitations, a new imaging modality is needed to enable functional *in vivo* imaging at cell-scale resolution deep in living animals. This would allow imaging of millions of cells in intact tissues at single cell resolution so that spatiotemporal aspects of biological processes could be studied from their functional bases in fine anatomical context.

Optical Coherence Tomography (OCT) uses low-coherence interferometry to provide micron-scale spatial resolution and millimeter-scale depth of penetration^9^, making it a potential candidate to fill the current gap in functional imaging technology. To date, applications of OCT have focused on studies of tissue structure or blood flow and oxygenation^10^,^11^,^12^. Several studies have attempted to adapt OCT as a molecular imaging modality by using a variety of nanoparticles, most commonly gold nanorods (GNRs)^13^,^14^. However, the total optical extinction of these conventional particles is dominated by absorption rather than scattering^15^, leading to poor OCT contrast. Furthermore, these studies have been performed largely *in vitro*, where phantom background scattering is significantly reduced relative to that of real tissues. The greatest challenge for realizing OCT as a contrast-enhanced modality for molecular imaging is to achieve signal from exogenous agents that can overcome the intrinsic signal of living tissues.

GNRs are generally good candidates for *in vivo* imaging because they exhibit plasmonic properties within the near-infrared “biological imaging window^16^”. However, the relatively small GNRs (~50 × 15 nm) commonly used in biomedical imaging do not scatter enough light to overcome the endogenous tissue signal detected by OCT. Larger GNRs with greatly enhanced scattering cross-sections^17^,^18^ could potentially achieve the signal-to-background ratio necessary to realize the advantages of OCT with molecular contrast. However, such particles have only recently been explored for biological applications, such as mapping constrained diffusion in 3D cell culture^19^. The current lack of high-contrast imaging agents has impeded the development of OCT as a functional molecular imaging technique. Consequently, the technological gap that exists between low-resolution/high-penetration and high-resolution/low-penetration imaging modalities has persisted.

Here we demonstrate functional imaging with contrast-enhanced OCT *in vivo*. To achieve this goal, we implemented large GNRs (~100 × 30 nm, hence called LGNRs) that were adapted for biological use as OCT contrast agents as recently reported^20^. We detected these agents *in vivo* by implementing a broadband Spectral Domain OCT (SD-OCT) system and custom spectral algorithms. LGNRs produced ~110-fold greater spectral signal per particle than conventional GNRs. We demonstrated an *in vivo* sensitivity of at least 250 pM LGNRs, and we noninvasively imaged through intact skin to see small blood vessels (~20 μm in diameter) up to 750 μm deep in tumor tissue. We also showed the functional capabilities of our approach by observing spatial patterns of fluid drainage in mouse ear lymphatic networks. In addition to providing high-resolution maps of lymph vasculature, contrast-enhanced OCT images were used to determine the locations and functional states of lymphatic valves that maintain unidirectional lymph flow, which is a key component of proper immune function in living animals.

Through the use of improved OCT contrast agents as well as custom spectral detection and processing methods, we were able to acquire images containing both rich anatomical structure and insight into functional biology at high spatial resolution. The high scan rates of SD-OCT also enable monitoring of physiological processes on timescales as short as a few seconds over millimeter fields of view. Based on its current capabilities, our contrast-enhanced OCT method, which we call MOZART, provides an ideal platform for *in vivo* targeted molecular imaging studies.

## Results

**Figure 1:**
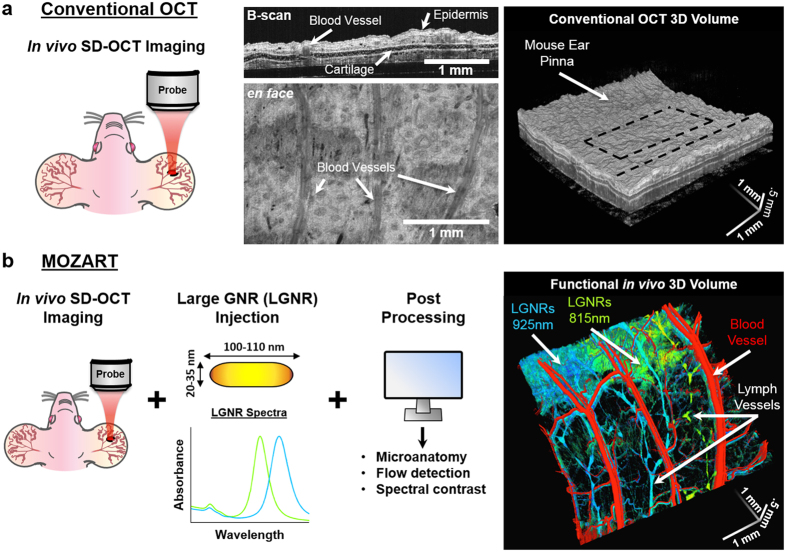
Overview of MOZART and its *in vivo* imaging capabilities. (**a**) Conventional OCT scan of the mouse pinna shows micro-anatomic structures in 2D (B-scan), 2D *en face* slice, and volumetric rendering. The dashed lines on the volumetric rendering of the OCT structure show the locations of the B-scan and *en face* slice. (**b**) MOZART combines SD-OCT with large GNRs (LGNRs) as contrast agents that are detected with custom adaptive post-processing algorithms. This approach can be used to create images that contain additional functional information *in vivo*. The MOZART image reveals subcutaneously-injected LGNRs with two different spectra (green and cyan) draining into lymph vessels as well as flow in blood vessels (overlay in red). The conventional OCT and MOZART 3D images depict the same region (each volume is 4 mm × 4 mm × 1 mm).

MOZART is based on a spectral domain OCT (SD-OCT) with a broad superluminescent diode (SLD) source and a spectrometer imaging at wavelengths from 800–1000 nm. We enhanced the contrast of this SD-OCT by using LGNRs with tuned resonance peaks and post-processing algorithms that were tailored to detect LGNRs in living tissue. We demonstrated that MOZART complements conventional OCT by imaging the ear pinnae of living mice in two types of experiments. In the first type, we imaged blood vessels in tumor and healthy tissue before and after intravenous (IV) injection of LGNRs. In the second type, we imaged LGNR drainage into lymph vessels following subcutaneous injections (Fig. 1).

### Post processing algorithms

**Figure 2:**
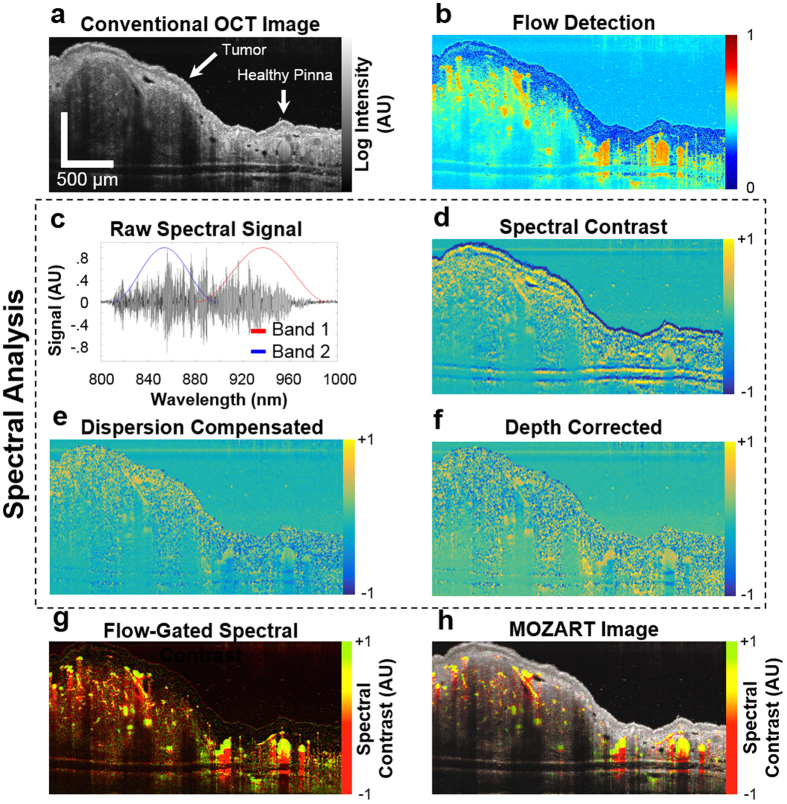
Description of post processing steps and results. (**a**) OCT log intensity image, showing the structure of a tumor on the pinna after IV injection of LGNRs. (**b**) Result of the flow detection algorithm. The image includes vertical shadowing below the vessels, which is a typical artifact of speckle variance methods for detecting flow. (**c**) The recorded interferogram is divided into two bands for implementation of a dual-band approach to detect LGNRs. The black line shows the interferogram after the subtraction of the spectrum of the SLD. The red and blue lines show the Hann filters used to window the interferogram. (**d**) Logarithmic representation of the spectral contrast. (**e**) As in (**d**), after iterative dispersion compensation. (**f**) As in (**e**), after applying depth-dependent gain to the bands to compensate for depth-dependent spectral aberrations. (**g**) Flow detection (intensity) combined with the spectral contrast (hue) to create a spectral map of the blood vessels in the tumor following LGNR injection. LGNRs in the blood vessels are shown in yellow-green. Regions below LGNRs appear red due to the spectral neutrality of the tissue below the blood vessels. (**h**) As in (**g**), with the inclusion of OCT intensity signal in gray scale to show tissue anatomy. Scale bars are 500 μm.

OCT relies on low-coherence interferometry to detect the positions of scatterers within a sample^9^. In SD-OCT, the locations of scatterers are retrieved by performing a Fourier transform on interferograms obtained from imaging with a broadband illumination source^21^. This standard approach produced images with purely structural information (Fig. 2a). We developed custom processing algorithms (Supplementary Fig. S1) based on a previously-described dual-band method^22^ to divide raw SD-OCT interferograms into two spectrally-distinct subsets, denoted here as Band 1 and Band 2. These bands correspond roughly to wavelength ranges of 900–1000 nm and 800–900 nm, respectively (see *Methods*) (Fig. 2c). The interferograms of each band were reconstructed to create OCT images in the spatial domain. These two images were subtracted and then divided by the OCT image reconstructed from the full interferogram. We define the difference between the reconstructed bands as spectral signal and the normalized difference between the reconstructed bands as the spectral contrast (Fig. 2d). Mere subtraction of the images reconstructed from the two halves of the spectrum was insufficient for recreating a reliable image of the spectral information in the sample. This was due to several spectral effects that occur in all OCT systems: dispersion, chromatic aberrations from optics and signal processing, and absorption.

In OCT, dispersion is caused by path length mismatch between the sample and reference arms when light travels through dispersive media (i.e., materials that have unequal refractive indices across wavelengths). Dispersion can be caused by optical elements in the OCT system and the sample itself. Dispersion compensation can be performed during post-processing image reconstruction by adding a phase that has a square dependence on the wavenumber. The coefficient in the exponent of this phase term is typically chosen to maximize image sharpness. Because we can assess dispersion by the decorrelation of the two reconstructed bands, we developed an adaptive method to choose the coefficient that minimizes band decorrelation (Fig. 2e).

Next, we observed that the reconstructed difference image contained depth-dependent spectral artifacts, although tissue is in general spectrally neutral. This dependence is caused primarily by three effects. First, the sampling of light by the spectrometer causes a “roll-off” effect in which the OCT intensity decreases with depth. The spectrometer pixel is uniform in wavelengths and thus non-uniform in wavenumbers, which causes roll-off to be spectrally dependent. Second, the optical setup introduces chromatic aberrations near the focal plane. This was the most dominant effect in our images. A third source of spectral artifacts was spectral absorption caused by the sample and the optical setup. The artifacts produced by these three effects were compensated by assuming a neutral region in the image and calculating an approximate depth-dependent gain. We applied an algorithm for automatic tissue detection to perform efficient compensation (Fig. 2f), which was critical for processing large datasets. Regions of static tissue suffered from significant spectral speckle noise due to the different speckle patterns in each band. However, LGNR movement (for example, by diffusion or circulation in blood) allowed temporal averaging of the speckle pattern to achieve a clear spectral signal. Thus for *in vivo* experiments, in which speckle dominated static regions of tissue, the spectral contrast was “flow-gated.” Regions of flow were detected by measuring speckle variance^23^ and applying compensation for low signal, which was able to reveal blood vessels deep in tissue (Fig. 2b).

We incorporated flow detection (depicted as image intensity) with the spectral contrast (depicted as image hue) to yield flow-gated images of spectral contrast in blood vessels (Fig. 2g). Flow and spectral contrast were also combined with tissue structure obtained by straightforward reconstruction of the OCT interferogram (in this case, spectral contrast, flow detection, and OCT intensity encoded image hue, saturation, and value, respectively). This reconstruction allowed simultaneous visualization of spectral and functional information in anatomical context (Fig. 2h).

### Application of LGNR contrast agents for OCT

**Figure 3:**
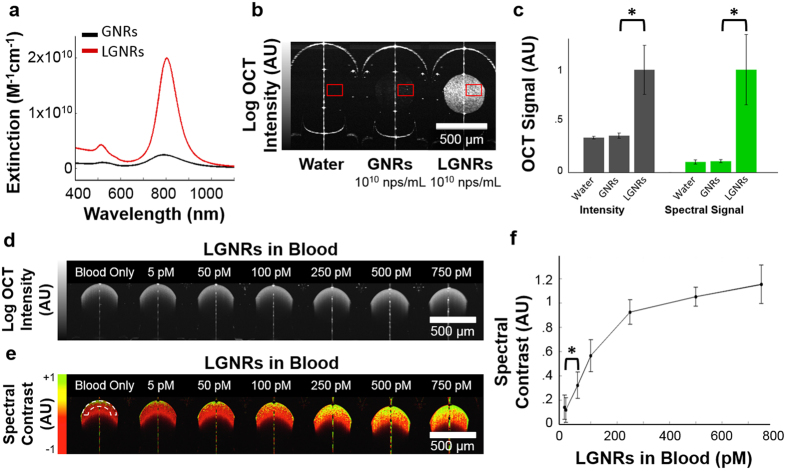
Evaluation of GNR and LGNR contrast. (**a**) LGNRs exhibit ~8-fold greater total extinction than conventional GNRs at equal nanoparticle concentration (nps/mL). GNRs and LGNRs are shown in black and red, respectively. (**b**) When prepared to equivalent particle concentrations (1 × 10^10^ nps/mL), LGNRs exhibit significantly greater OCT signal than GNRs. At this concentration, GNRs are barely visible above the noise threshold of the system. GNRs were imaged inside circular glass capillary tubes that exhibit strong specular reflections, shown as high signal vertical line artifacts. (**c**) Quantitative analysis of the regions outlined in (**b**) shows that LGNRs exhibit ~30-fold greater OCT intensity and ~110-fold greater spectral signal than GNRs per particle (*p < 0.001 in each case). The noise level was subtracted from all values. (**d**) Log OCT intensity of increasing concentrations of LGNRs in blood. (**e**) The spectral contrast of samples in (**d**) without depth-dependent compensation, showing the increase in spectral hue from red to yellow-green as LGNR concentration increases. The dashed line shows the region that was analyzed for quantification of spectral contrast. (**f**) Quantification of spectral contrast of LGNR concentrations in blood. Each measurement is the average spectral contrast over 4 A-scans within analyzed regions in (**e**). These regions were selected to reduce the effect of absorption on the spectral contrast. We are able to detect LGNRs at concentration as low as 50 pM (*p < 0.001). Scale bars are 500 μm. Quantitative data are presented as mean ± SD.

GNRs are commonly-used contrast agents that are typically ~50 × 15 nm in longitudinal and axial dimensions, respectively. While GNRs of this size are excellent absorbers of near-infrared light, they are not efficient scatterers. Moreover, these GNRs produce more forward scattering than backscattering and thus are poor OCT contrast agents. Recently, a method for producing much larger GNRs (LGNRs) was developed^18^. Using this method, we synthesized LGNRs that were ~100 × 30 nm in size as measured by transmission electron microscopy (TEM) (Supplementary Fig. S2). LGNRs exhibited a peak longitudinal surface plasmon resonance (LSPR) at 815 nm and displayed an ~8-fold greater extinction coefficient than conventional GNRs of equivalent plasmonic resonance (Fig. 3a). A separate batch of LGNRs with 925 nm LSPR was also prepared (Supplementary Fig. S2) for multiplexed studies.

While light scattering intensity is a critical consideration for *in vivo* OCT agents, biostability and non-toxicity are also vital. To achieve these characteristics, GNRs are commonly coated with thiolated poly(ethylene glycol) (PEG-SH) reagents^24^. We recently found that LGNRs coated with methoxy-PEG-SH (MW ~5 kDa) did not remain stable through the numerous washing steps required for biological use. However incubation of LGNRs with poly(sodium 4-styrenesulfonate) (PSS, MW ~70 kDa) produced particles (LGNRs-PSS) that were robustly stable in both DDI H_2_O and FBS^20^. Previous reports have shown that PSS is safe for *in vivo* use^25^,^26^. For the purposes of this study, we further coated LGNRs-PSS with methoxy-PEG-SH and washed them to produce LGNRs-PSS-mPEG to ensure biocompatibility (Supplementary Fig. S4a). LGNRs-PSS-mPEG were used in all lymph imaging experiments. We also separately prepared LGNRs-PSS coated with non-specific IgG1-derived antibodies (hence called LGNRs-Ab) to show that LGNRs-PSS present a broad platform that can be readily modified with antibodies for future molecular targeting experiments. Briefly, LGNRs-PSS were incubated with a heterobifunctional Biotin-PEG-SH (MW ~5 kDa) and washed to produce LGNRs-PSS-PEG-Biotin (Supplementary Fig. S4b). These particles were sequentially incubated with NeutrAvidin and biotinylated Mouse IgG1 Isotype antibody and washed (See *Methods*). Intravenous injections of LGNRs-PSS-mPEG or LGNRs-Ab (up to 250 μL of 8.5 nM) did not induce signs of distress in mice observed over a period of at least four weeks.

To demonstrate the clear advantages of LGNRs for contrast-enhanced OCT, we compared the OCT signals of LGNRs and GNRs *in vitro*. LGNRs and GNRs with equivalent LSPR were used for accurate comparison. LGNRs produced ~30-fold greater OCT intensity per particle than GNRs. The use of spectral detection resulted in an additional improvement to LGNR signal such that LGNRs exhibited ~110-fold greater spectral signal than GNRs on a per particle basis (p < 0.001, Fig. 3b,c). A portion of the increased scattering from LGNRs was due to their greater cross-sectional area. When prepared to equivalent Au mass concentration (mg/mL) to account for differences in the number of Au atoms per particle, LGNRs exhibited ~3.5-fold greater OCT intensity than GNRs (p < 0.001) due to greater proportional backscattering (Supplementary Fig. S5). Accounting for the additional enhancement from spectral detection, this indicates that LGNRs produce ~13-fold greater spectral OCT signal than GNRs on an equivalent mass basis.

Using our spectral processing algorithms, we were able to detect single LGNRs in aqueous solutions in glass capillary tubes. The ability to detect single LGNRs was evidenced by the fact that numbers of observed bright puncta in OCT images were consistent with expected numbers of individual particles for the given LGNR concentration in the imaged volumes. Additionally, the spectral contrast of these puncta was consistent with that expected from single LGNRs (Supplementary Fig. S6a,b).

### *In vitro* imaging sensitivity

We next characterized our system’s ability to detect the spectral characteristics of LGNRs *in vitro*. Spectral analysis methods detected LGNRs with 50 pM sensitivity (p < 0.001) in freshly-collected whole rat blood, indicating that LGNRs easily produced OCT spectral contrast that was distinguishable in biological backgrounds (Fig. 3d–f, Supplementary Fig. S7). Using non-intensity-normalized spectral contrast provided slightly different contrast-enhanced images (Supplementary Fig. S7). Non-intensity-normalized images can allow better relative quantification of LGNRs in blood owing to their intrinsic enhanced scattering. However, this signal is more sensitive to intensity variations such as those caused by roll-off, distance from the focal plane, and absorption.

### *In vivo* LGNR sensitivity and circulation time

**Figure 4:**
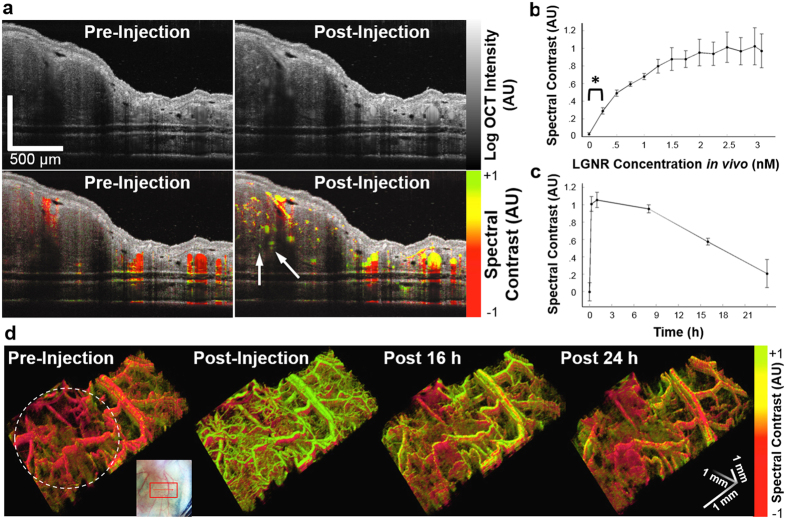
Contrast enhancement of OCT images of an *in vivo* tumor model. (**a**) OCT log intensity images (top) and combined spectral contrast images (bottom), before (left) and after (right) IV injection of LGNRs-Ab. LGNRs-Ab are detected in the blood vessels, which are shown in red before the injection and appear yellow-green after injection due to LGNR-Ab spectral signal. LGNRs-Ab enhance the ability to see small blood vessels deep in the tumor (white arrows). Scale bars are 500 μm. (**b**) Spectral signal in the blood vessels during an incremental injection of LGNRs-Ab IV. Measurements were collected from three mice and three blood vessels per mouse. We are able to detect LGNRs-Ab from the first incremental injection, which is equivalent to a LGNR-Ab concentration of 250 pM in the blood (*p < 0.001). (**c**) Spectral signal in the blood vessels before and up to 24 h after IV injection of LGNRs-Ab. Measurements were collected from three mice and three blood vessels each. (**d**) 3D volumes (4 mm × 2 mm) of spectral contrast signal in blood vessels of tumor (circled by dashed white line) and adjacent healthy tissue before and 0, 16, and 24 h after LGNR-Ab injection. The color scale is the same as in (**a**). The inset shows 2D and 3D scan locations on top of a photograph of the mouse ear. Quantitative data are presented as mean ± SEM.

We demonstrated our ability to detect LGNRs *in vivo* by imaging a mouse tumor xenograft model. Female nude (nu^−^/nu^−^) mice were inoculated with 1–2 × 10^6^ U87MG cells in the right ear pinna. Seven days after tumor cell injections, we injected LGNRs-Ab (250 μL of 23.5 nM) in 20 μL increments IV into the mice (N = 3) to quantify LGNR-Ab spectral signal sensitivity *in vivo*. Cross-sectional OCT images were acquired after each incremental injection, and spectral contrast in blood vessels (N = 3 for each mouse, see *Methods*) was measured. While blood vessels initially exhibited neutral spectral signal, strong spectral bias toward the LGNR-Ab peak resonance was observed in these vessels following the full LGNR-Ab injection (Fig. 4a). Spectral analysis enabled detection of LGNRs-Ab at concentrations as low as 250 pM (p < 0.001) in mouse ear vasculature (Fig. 4b, Supplementary Fig. S8). We next characterized the pharmacokinetics of LGNRs-Ab in IV-injected mice (N = 3). LGNRs-Ab exhibited a spectral contrast circulation half-life (t_1/2_) of ~18 h based on spectral quantification over a 24 h period post-injection (Fig. 4c). This circulation profile suggested that antibody-modified LGNRs are attractive candidates for future molecular targeting studies^27^.

### Imaging LGNRs in tumors

OCT enables higher resolution than photoacoustic microscopy^5^ and deeper imaging over larger fields of view than multiphoton microscopy^7^. With the addition of contrast enhancement, these advantages make OCT a powerful tool for the noninvasive study of tumor growth and morphology. Wide-field (4 mm × 2 mm) volumetric OCT images showed differences in blood vessel morphology between healthy ear tissue and tumor tissue prior to LGNR-Ab injection while the signal in both healthy and tumor vasculature is spectrally neutral. A dramatic increase in spectral contrast was observed following LGNR-Ab injection in healthy and tumor vasculature. Many additional small blood vessels became visible owing to increased backscattering from LGNRs-Ab, especially within the tumor tissue (Fig. 4d, Supplementary Fig. S8–S10).

### Imaging functional lymph drainage with LGNR multiplexing

**Figure 5:**
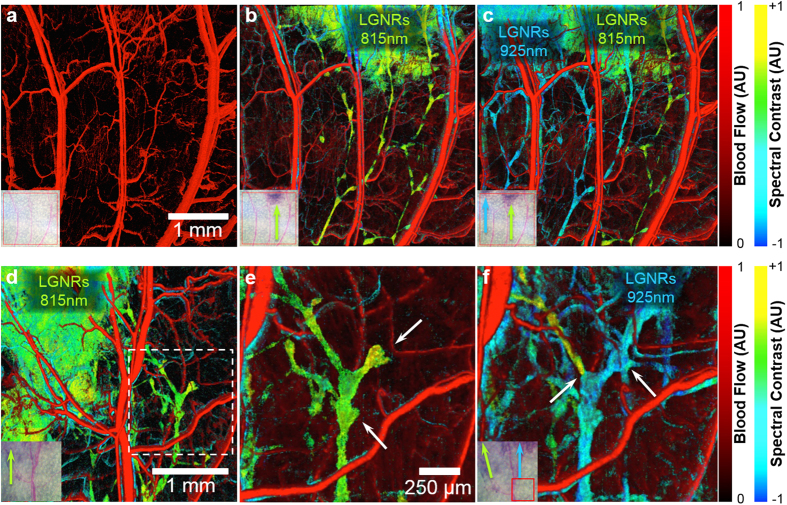
Study of LGNRs-PSS-mPEG draining into lymph vessels of the pinnae *in vivo*. (**a**) *En face* view of flow detection, showing the blood vessels (in red) prior to injection. The inset is a photograph of the ear showing the scanned region. (**b**) The spectral contrast showing the first injection of 815 nm LGNRs (shown as green) with an overlay of the blood vessels from (**a**). (**c**) As in (**b**), after the second injection, of 925 nm LGNRs (shown as cyan-blue). The LGNRs are filling the lymph vessels and draining from the injection sites at the edge of the ear (top of image) to the base of the ear (bottom of image). (**d**) *En face* view of a separate mouse after an injection of 815 nm LGNRs at the left side of the image. (**e**) A close-up view of the region marked by a dashed line in (**d**), showing a junction in the lymph network. The location of valves between adjacent lymphangions can be clearly observed (white arrows). One lymphangion appears to have a dead-end, indicating the location of a valve and the lack of bidirectional LGNR flow. (**f**) The same region as in (**e**), after an injection of 925 nm LGNRs at the right side of the image. Both 815 nm and 925 nm LGNRs reach the lymphatic junction. The lymph vessel on the left side of the junction still contains 815 nm LGNRs (green) while a previously-unseen vessel on the right side and the vessel downstream of the junction show 925 nm LGNRs (cyan-blue). Unidirectional lymph flow is evidenced by both the abrupt separation of 815 nm and 925 nm LGNRs at the left side valve junction and the full right side vessel upstream of the previously-observed dead end after the 925 nm LGNR injection (white arrows).

Previous studies have used OCT to identify networks of lymphatic vessels by their lack of scattering contrast^10^; however these methods do not provide information on functional lymph flow within vessels, such as the areas of tissue that they drain or lymph flow directionality. We used MOZART to image the spatial dependence of fluid drainage in the initial lymphatics of mice pinnae (N = 3). Flow-gated OCT images acquired prior to any injection depicted blood vasculature (Fig. 5a, Supplementary Fig. S11a). Immediately following injection of 815 nm LGNRs-PSS-mPEG (2 μL of 8.5 nM), we observed an extensive network of draining lymph vessels with diameters as small as ~20 μm (Fig. 5b, Supplementary Fig. S11b). After 30 minutes, we injected a separate dose of spectrally-distinct 925 nm LGNRs-PSS-mPEG (2 μL of 8.5 nM) ~1 mm away from the first injection site. The two LGNR types were distinguished from each other using spectral analysis. We observed several previously-unseen lymph vessels draining the 925 nm LGNR injection site. Interestingly, several lymph vessels that exhibited strong 815 nm LGNR signal after the first injection showed 925 nm LGNR signal or mixed LGNR signal after the second injection. Some lymph vessels containing mixed spectral signals exhibited areas of discrete spectral contrast rather than an average of the two LGNR spectra (Fig. 5c, Supplementary Fig. S11c).

A critical aspect of healthy lymph vessel function and mammalian immunity is unidirectional drainage of interstitial fluids and cell trafficking to lymph nodes^28^,^29^. Unidirectional flow is maintained in initial lymphatic vessels through fluid pressure and the function of valves present at each end of the lymphangion segments that compose the lymph vasculature^30^,^31^. MOZART was capable of distinguishing individual lymphangions in actively-draining vessel networks (Fig. 5d,e). Sequential injections of multiplexed LGNRs-PSS-mPEG also allowed inference of the functional states of select lymphatic valves that maintained unidirectional drainage. The effects of unidirectional valve function were particularly obvious at points where separate lymph vessels that drained LGNRs-PSS-mPEG with distinct spectra joined into the same downstream collecting vessel (Fig. 5f).

## Discussion

In summary, we have developed a contrast-enhanced OCT imaging platform called MOZART that enables functional biological imaging studies in small living subjects. We have demonstrated LGNRs as readily-functionalized, high-sensitivity imaging agents and developed adaptive spectral processing algorithms to enable aberration-free, contrast-enhanced *in vivo* studies with OCT. Using this approach, we were able to detect 250 pM LGNRs in circulation following intravenous injection into living mice. Considering our image voxel size is 4 μm × 8 μm × 8 μm, this translates to an unprecedented sensitivity of 40 particles per voxel. By comparison, most other modalities including photoacoustic contrast or fluorescence imaging require at least 1000-fold more contrast particles per imaging voxel^2^,^32^. We used the enhanced scattering of LGNRs to image small capillaries up to 750 μm deep in U87MG tumor xenografts. The contrast enhancement achieved with our method also highlighted differences in vascular morphology between healthy ear and tumor tissue. Furthermore, we demonstrated multiplexed detection of LGNRs, which enabled visualization of active drainage in initial lymphatics at different locations within the mouse ear and in some cases allowed us to observe the functional states of individual lymphangions and valves in initial lymphatic networks.

Spectral methods have been used previously to detect GNRs, however these experiments were performed in aqueous solutions and phantoms that lacked intrinsic scattering from blood and other tissues^14^,^33^. Another study detected large pools of concentrated plasmonic nanoparticles following superficial intradermal injections^34^. However images produced by this method had limited spatial resolution and lacked flow detection, thus preventing observations of blood or lymphatic vasculature structure and function. Furthermore, spectral images created in each of these studies lacked the combination of sample-specific dispersion compensation and adaptive depth-dependent correction, each of which is critical for obtaining accurate spectral images that are free of chromatic aberrations. Without proper compensation, GNRs with uniform resonance exhibit varying spectral signal at different axial depths within OCT images. This spectral non-uniformity hinders accurate GNR identification, especially in the context of background tissue scattering. The need for depth-related compensations was addressed in the past^22^, however, we found that the non-adaptive compensation that has been previously described was insufficient for spectral imaging as it created additional artifacts in images of living tissues. Therefore, we developed an adaptive algorithm package that is able to correct for dispersion and depth-related aberrations for every image. These post-processing steps proved to be essential for obtaining reliable high-resolution spectral contrast *in vivo*. Such corrections are critical for the ability to consistently and easily identify nanoparticles with unique scattering spectra at a high sensitivity.

Conventional small GNRs have also been explored as OCT contrast agents using non-spectral detection methods. One study successfully distinguished GNRs from tissue scattering phantoms through detecting changes in light polarization due to particle Brownian motion, but no *in vivo* experiments were reported^13^. Other studies have used photothermal OCT (PT-OCT) to detect GNRs by absorption^35^,^36^. However, PT-OCT exhibits limited multiplexing capabilities compared to SD-OCT approaches, and strong absorption by blood produces additional obstacles for the detection of PT-OCT contrast agents. To date, these and other limitations including slow scan speeds have posed challenges for developing OCT as a contrast-enhanced functional imaging technique.

The work described herein used LGNRs with enhanced scattering properties to enable strong OCT contrast *in vivo*. Consistent with theory, LGNRs exhibited greater scattering (~30-fold greater per particle) than conventional GNRs of similar plasmonic resonance^15^,^17^. Importantly, the use of spectral detection methods enabled an even greater signal enhancement from LGNRs (~110-fold more signal than GNRs) when prepared in concentrations for which OCT signal is linear^37^. We further developed NeutrAvidin-coated LGNRs that can be adapted with biotinylated ligands of interest (such as monoclonal antibodies) for future molecular targeting studies. We also characterized LGNR-Ab circulation properties to inform future *in vivo* applications. Furthermore, LGNRs can be synthesized in many spectral “flavors” ranging from 600 to 1000 nm LSPR. As described in this work, future MOZART studies will employ LGNRs of distinct resonances prepared with different targeting biomolecules to enable spectrally multiplexed molecular imaging studies.

OCT has found recent success in filling the gap between micrometer resolution and millimeter depth of penetration imaging techniques. Label-free OCT techniques have been used for vascular imaging including blood oxygenation and flow quantification^10^,^11^,^12^. Algorithmic approaches have also been developed to identify the presence of lymphatic vessels^10^,^38^. MOZART offers additional OCT capabilities including greater achievable signal in small vessels deep within tissue and functional imaging of interconnected networks of draining lymphatics. Spectral detection of LGNRs affords excellent imaging sensitivity, which is vital for detecting specific protein targets in live animal models of disease and development. Future experiments will implement broader illumination sources and many distinct LGNRs with wide spectral separation to achieve greater multiplexing capabilities. Molecular targeting capabilities of LGNRs will also be demonstrated and rigorously characterized.

The work described in this report realizes new functional capabilities for *in vivo* OCT imaging and establishes MOZART as an OCT platform for *in vivo* molecular studies. This platform can be used for pre-clinical studies of molecular tumor heterogeneity, lymphangiogenesis, and disruption of lymphatic networks in lymphedema. Since OCT instruments dedicated for imaging the retina exist, MOZART could also be used to study retinal diseases such as age-related macular degeneration at the molecular scale in living animals^38^,^39^. Future clinical applications include skin and mucosal imaging as well as intraoperative imaging of surgically-exposed tissue.

## Methods

### GNR synthesis and characterization

LGNRs (~100 × 30 nm) were synthesized by adapting a previously described method^18^. Briefly, 9.0 g of cetyltrimethylammonium bromide (CTAB, ≥98%, TCI, CAS# 57-09-0) and 1.234 g of sodium oleate (NaOL, ≥97%, TCI, CAS# 143-19-1) were dissolved in 250 mL DDI H_2_O at 50 °C with 400 rpm stirring. Once dissolved, the temperature was decreased to 30 °C and 50 mL of 0.004 M silver nitrate (AgNO_3_, 99.9999% trace metal basis, Aldrich, CAS# 7761-88-8) was added. This solution was left undisturbed for 15 minutes, after which 250 mL of 0.00086 M gold chloride trihydrate (HAuCl_4_ • 3H_2_O, ≥49.0% Au basis, Sigma-Aldrich, CAS# 16961-25-4) was added. This mixture (the growth solution) was stirred for 90 minutes at 700 rpm, during which time the yellow-gold solution gradually turned colorless. A seed solution was prepared during this time from 5 mL of 0.2 M CTAB, 5 mL of 0.00043 M HAuCl_4_ • 3H_2_O, 0.6 mL of 0.01 M sodium borohydride (NaBH_4_, ≥99%, Fluka, CAS# 16940-66-2), and 0.4 mL of DDI H_2_O. The seed solution was aged for 30 minutes prior to use. 90 minutes after adding HAuCl_4_ • 3H_2_O to the growth solution, 2.1 mL of 12.1 N hydrochloric acid (HCl, Fisher, CAS# 7647-01-0) was added and the solution was stirred at 400 rpm for 15 minutes. 1.25 mL of 0.064 M L-ascorbic acid (reagent grade, Sigma, CAS# 50-81-7) was then added, and the growth solution was stirred at 1200 rpm for 30 seconds. Finally, 0.1 mL of the aged seed solution was added to the growth solution and stirred for 30 seconds at 1200 rpm. The mixture was left at 30 °C without stirring for 12 h to allow LGNR formation. Visible and near-infrared absorbance spectra were measured for each LGNR batch using a Cary 6000i spectrometer. The method described above produced LGNRs that exhibited peak Longitudinal Surface Plasmon Resonance (LSPR) at 815 nm and a spectral Full Width at Half Maximum (FWHM) of 100 nm. The synthesis was repeated using 25 mL of 0.0004 M AgNO_3_, 1.5 mL HCl, and 0.05 mL seed to produce a second batch of LGNRs with peak LSPR at 925 nm and a spectral FWHM of 120 nm. LGNRs were characterized using a JEOL TEM 1400 electron microscope to determine particle morphology and size distributions. LGNRs with LSPR = 815 nm were 107 ± 7 nm in length and 32 ± 2 nm in width (N = 20). LGNRs with LSPR = 925 nm were 105 ± 7 nm in length and 22 ± 1 nm in width (N = 20). Conventional GNRs (~50 × 15 nm, LSPR = 800 nm) were synthesized using an original method by Nikoobakht and El-Sayed^40^. Conventional GNRs were used for OCT signal comparisons with LGNRs *in vitro*.

### GNR Functionalization

After synthesis, LGNRs were centrifuged at 2550 × g and resuspended to 1 nM LGNRs (OD 20 at peak λ). Then, LGNRs were reacted with 100 μm poly(sodium 4-styrenesulfonate) (PSS, MW ~70 kDa, Aldrich, CAS# 25704-18-1) for 5 minutes with vortexing to produce LGNRs-PSS. LGNRs-PSS were then centrifuged at 2550 × g and resuspended to 1 nM. This PSS coating process was performed a total of three times to ensure complete polyelectrolyte coating of the LGNR surface. Washed LGNRs-PSS were then incubated with 1 mg/mL methoxy-poly(ethylene glycol)-thiol (mPEG-SH, MW 5 kDa, Laysan Bio, Arab, AL) for 24 h at room temperature based on a previous method^24^ to produce LGNRs-PSS-mPEG. Separate batches of LGNRs-PSS-mPEG at each LSPR (815 nm and 925 nm) were washed twice using the same centrifugation conditions and concentrated to 8.5 nM (OD 170 at peak λ) for use in subcutaneous injections.

Separately, LGNRs-PSS with 815 nm LSPR were incubated with Biotin-PEG-SH (MW ~ 5 kDa, NANOCS, PG2-BNTH-5k) and washed to produce LGNRs-PSS-PEG-Biotin. 1 mL of 1 nM LGNRs-PSS-PEG-Biotin was then incubated with 200 μL of 1 mg/mL NeutrAvidin (NA, Thermo Scientific, #31000) to produce LGNRs-PSS-PEG-Biotin-NA. These particles were prepared in a 10 mL glass scintillation vial and washed once by centrifugation at 1500 × g for 30 minutes. Washed LGNRs-PSS-PEG-Biotin-NA were then incubated with 5 μL of 0.5 mg/mL Mouse IgG1 Biotin Antibody (eBioscience, Clone P3.6.2.8.1) to produce LGNRs-Ab. LGNRs-Ab were prepared to 23.5 nM (OD 470 at peak λ) for use in intravenous injections.

### OCT System

All OCT images were acquired using a Ganymede High-Resolution SD-OCT system (ThorLabs, Newton, NJ). The light source is a SLD with a center wavelength of 900 nm and a 200 nm full bandwidth (Δλ = 800-1000 nm), which provides 2.1 μm axial resolution in water. The spectrometer acquires 2048 samples for each A-scan at a rate of 30 kHz. At the beginning of each acquisition, the OCT is programmed to measure the spectrum of the SLD 25 times. This measurement was used for the reconstruction of the OCT signal. All images excluding lymph experiments were acquired using a lens that provides a lateral resolution of 8 μm (FWHM) and depth of field (DOF) of 143 μm in water (LSM03-BB, ThorLabs, Newton, NJ). Lymph experiment images were acquired using a lens that provides a lateral resolution of 4.2 μm (FWHM) and DOF of 32 μm in water (LSM02-BB, ThorLabs, Newton, NJ). During *in vivo* imaging, we optimized light transmission to the sample by applying ultrasound (US) gel to the mouse skin and covering the gel with a one sided anti-reflective (AR) coated glass, with the coating at the air-glass interface. Additionally, we used double-sided tape to attach the ear of the mouse to a mount in order to minimize movement artifacts. All 2D images were created from 100 consecutive B-scans, although sufficient spectral contrast and signal-to-noise ratio (SNR) are observed even with 10 B-scans. The pixel spacing in the 2D scans is 2 μm and the total acquisition time for a 3 mm scan is approximately 5 s. The 3D images were created from 8 and 20 consecutive B-scans in the tumor and lymph drainage experiments, respectively, with 5 μm spacing in both axes. To improve SNR in the tumor volumes only, 5 scans were averaged so that a total of 40 scans were used for the post-processing. Next, the volumes were resampled at 5 μm spacing in all three axes. The volumes in Figs 4d and 5 were created by stitching 1 × 2 and 4 × 4 smaller volumes, respectively (resulting in total acquisition times of 1.5 and 7 minutes). Owing to the stability of the setup, registration was not needed; the volumes were simply concatenated.

### Post processing algorithms

All of our code was written and processed in Matlab 2014a and 2014b. Reconstruction of the OCT interferograms was done by first subtracting the DC term, which was acquired separately for every scan by our commercial system. Next we applied a non-equispaced discrete Fourier Transform^41^, which was implemented with matrix multiplication. The absolute value of this is the typical OCT intensity signal.

When performing spectral contrast image reconstruction, we divided the interferogram by the spectrum of the source (the DC term) in order to compensate for spectral imbalances originating from the SLD. The denominator was clipped from the bottom at a value of 10% of the average signal to avoid dividing by small numbers that correspond to noise.

The sizes of the two spectral bands used to extract the spectral contrast were chosen so that the reconstructed images had equal axial resolution. From the dependence of OCT resolution on the wavelength and bandwidth^42^ we determined that the high-wavelength band should contain 1124 samples while the low-wavelength band should contain 900 samples. Using the dual band method reduces the axial resolution to 4 μm. We used Hann windows for cropping the spectral bands in order to avoid artifacts. Each B-scan was reconstructed independently and then averaged with the results from B-scans at a similar location. After reconstruction of the bands, we applied a 3 × 3 pixel median filter to reduce noise while retaining high contrast features. The median filter is applied to an image that is sampled every 2 μm, which is smaller than the optical resolution of the system, therefore, the resolution remains almost unchanged by this filter.

Dispersion compensation was performed by finding the coefficient of the quadratic phase term iteratively by minimizing the absolute difference between the reconstructed images of the two bands. Higher-order dispersion was not compensated; however, assuming that the sample and reference arms of the OCT were approximately dispersion matched initially and that the sample itself is thin enough to avoid introducing significant higher order dispersion, the second order compensation is sufficient. In the future, correcting for higher-order dispersion may be performed to improve the axial resolution of the spectral contrast. The iterative search was done by minimizing the change in the coefficient when the absolute difference decreased and by changing the sign of the step when it increased. This processed converged within 30 iteration steps. We found that the dispersion in our setup changed between experiments, owing to the varying amount of US gel we used on top of the mouse ear. However, dispersion did not change within a given experiment.

The adaptive depth-dependent compensation algorithm was an essential step for the success of our method in extracting the spectral data from *in vivo* OCT scans. In samples thicker than several tens of μm, depth-dependent chromatic aberration and “roll-off” create a false spectral signal that may dominate the signal of our contrast agent. In order to compensate for these effects, we used a spectrally-neutral region in our scans to calibrate the depth-dependent spectral shift and then applied this calibration throughout the image. The spectrally-neutral region was either selected manually or found automatically. In order to process large volumes and be invariant to the scanning lens aberrations, we applied the compensation to every B-scan. To do this efficiently, we used a tissue detection algorithm that used the static tissue in each B-scan as a neutral reference. The tissue was segmented from the background using morphological operators (dilation and erosion, for filling holes caused by low intensity regions and speckle noise) and connected components. To avoid compensation according to regions with a high concentration of LGNRs, we removed the regions of the blood and lymph vessels (which were detected by their flow) from the segmented tissue. We reconstructed the spatial image for each band and measured the average signal in each image as a function of depth. We fit the depth-dependent signal to a high-degree polynomial (degree ~ 5) to reduce noise, and used the fitted polynomial to calculate the gain needed for each image to compensate depth-dependent effects. Generally, as a function of depth, one image has a gain lower than 1, and therefore, noise is attenuated. For the other image, in which the gain is higher than 1, the background noise is increased. However, owing to B-scan averaging and median filtering, this noise is negligible to begin with. Furthermore, gain values typically range between 0.8 and 1.2. We then multiplied the images by the appropriate depth-dependent gain and subtracted them. We refer to the subtracted reconstructed bands normalized by the OCT intensity as the spectral contrast. The spectral contrast can be scaled as needed.

Flow detection was performed by calculating the speckle variance in 4 or 5 consecutive scans and averaging this value over the acquired scans. The speckle variance was divided by the OCT intensity to allow detection of blood vessels deep in tissue in a manner independent of absorption by tissue and blood.

The OCT structure, the spectral contrast, and the flow information were combined in an HSV scheme. See Supplementary Fig. S1 for a block diagram of the post-processing steps. Images were displayed using either Matlab (2014a or 2014b) or ImageJ 3D Viewer.

### Signal quantification and imaging sensitivity *in vitro*

LGNRs and GNRs with the same peak absorbance were prepared to equal optical density (OD 1) in glass capillary tubes (inner diameter = 0.4 mm). Note that at this OD, GNRs are at ~8-fold higher concentration (molarity and nps/mL) than LGNRs. Along with a tube filled with DDI H_2_O (used to measure the average noise baseline), OD 1 LGNRs were imaged with the Ganymede High-Resolution OCT system. This imaging approach was repeated for tubes containing GNRs and LGNRs prepared to equivalent nps/mL. For low-concentration samples of LGNRs in water, puncta were counted if they exhibited intensity greater than the noise baseline. The spectral imaging sensitivity of our system was characterized *in vitro* by preparing different concentrations of LGNRs in whole rat blood (heparin added to prevent coagulation). Separate capillary tubes were prepared with blood only and LGNRs at 5 pM, 50 pM, 100 pM, 250 pM, 500 pM, and 750 pM in blood. These samples were imaged, and spectral OCT images were produced using the algorithms described above.

### Animal models, cell culture and tumor models

Female nude mice (nu^−^/nu^−^ 6–8 weeks old, Charles River Labs, Wilmington, MA) were used for all imaging studies. For the tumor models, U87MG human glioblastoma (American Type Culture Collection) were cultured under standard conditions. After the cells were lifted, they were centrifuged at 300 × g for 5 minutes in order to concentrate them into a small volume. The U87MG tumor models were generated by subcutaneous injections of 1 × 10^6^ to 2 × 10^6^ cells in 5 μL of cell media in the center of the right ear pinna (1 injection per pinna). When the tumor volume reached 2–10 mm^3^ (typically 6–10 days post injection), the mice were used for tumor imaging experiments including *in vivo* sensitivity and circulation studies. OCT measurements of tumor and nearby healthy tissue in living mice were acquired before and up to 24 h after the IV injection. Healthy mice (with no tumors) were used for all lymph imaging experiments. All animal experiments were performed in compliance with IACUC guidelines and with the Stanford University Animal Studies Committee’s Guidelines for the Care and Use of Research Animals. Experimental protocols were approved by Stanford University’s Animal Studies Committee (APLAC protocol 27499).

### Imaging sensitivity and circulation for *in vivo* tumor model

Tumor-bearing mice (N = 3) were anesthetized by inhalation of 2% isoflurane and placed on a 37 °C heating pad during imaging experiments. The right ear pinna of each mouse was mounted flat using double-sided tape and covered with US gel and a cover glass with one-sided AR coating for optimized imaging. Pre-injection three-dimensional and cross-sectional OCT images of the tumor region and healthy ear tissue were obtained. LGNRs-Ab (250 μL of 23.5 nM, LSPR = 815 nm) were then administered via tail-vein injection incrementally (10 μL and 20 μL increments). After each incremental injection, LGNRs-Ab were allowed to circulate for 1 minute prior to cross-sectional image acquisition. These images were used to calculate our system’s *in vivo* LGNR-Ab imaging sensitivity by quantifying spectral signal before and after each injection increment and correlating spectral signal values to known concentrations of LGNRs-Ab in circulation. The total blood volume of a 25 gram mouse is ~2 mL, which was used to convert LGNR-Ab injection amounts into LGNR-Ab concentration in circulation. Three-dimensional and cross-sectional images were also acquired at 0, 1, 8, 16, and 24 h post-injection for all mice. Using the spectral quantification described above, these images were used to determine a spectral contrast circulation half-life (t_1/2_) of ~18 h for LGNRs-Ab.

### Lymph drainage experiments

Healthy mice (N = 3) were anesthetized by inhalation of 2% isoflurane and placed on a 37 °C heating pad during lymph imaging experiments. Right ear pinnae were prepared for imaging as described for tumor imaging experiments. Pre-injection three-dimensional and cross-sectional OCT images of ear were obtained. LGNRs-PSS-mPEG (2 μL of 8.5 nM, LSPR = 815 nm) were then subcutaneously injected near the outer edge of the ear pinna. Three-dimensional OCT images were acquired 15 minutes after this injection to visualize lymphatic drainage from the injection site. After this image acquisition, a second dose of LGNRs-PSS-mPEG with a distinct scattering resonance (LSPR = 925 nm) was injected ~1 mm away from the original 815 nm LGNR injection site. Three-dimensional OCT images were acquired 15 minutes after this second injection to provide spectrally-multiplexed maps of lymphatic drainage networks.

### Statistical methods

*In vitro* comparison of conventional GNRs vs LGNRs: After intensity image acquisition, regions of interest (~32,000 pixels each) were chosen at the same focal depth within each capillary tube to calculate mean OCT signals and standard deviations for each sample. These data were compared using two-tailed Student’s t-tests to determine statistical significance.

*In vitro* spectral sensitivity measurements: Quantification of the spectral signal from each tube was performed in a manner analogous to the method described for the conventional GNR and LGNR signal comparison (2000 pixels per ROI). These data were also compared using two-tailed Student’s t-tests to determine the spectral sensitivity of our system.

*In vivo* spectral sensitivity measurements: Sensitivity measurements were performed and calculated for each of the three mice individually. For each mouse, the spectral signals in three independent blood vessels within each cross-sectional image were analyzed. Sensitivity was measured as 250 pM, 500 pM, and 250 pM LGNRs for individual mice, respectively (p < 0.05, two-tailed Student’s t-test in all cases). We also calculated the sensitivity for the group of three mice using each mouse as an independent measurement, and we found the sensitivity for the group was 250 pM (p < 0.001, z-test).

## Additional Information

**How to cite this article**: Liba, O. *et al*. Contrast-enhanced optical coherence tomography with picomolar sensitivity for functional *in vivo* imaging. *Sci. Rep.* **6**, 23337; doi: 10.1038/srep23337 (2016).

## Supplementary Material

Supplementary Information
