# Supplementary material for: Contrast-enhanced optical coherence tomography with picomolar sensitivity for functional in vivo imaging
Source: Sci Rep. 2016 Mar 18;6:23337. doi: 10.1038/srep23337 (PMC4796912; doi:10.1038/srep23337)
Supplement: Supplementary Information [file srep23337-s1.pdf]

Supporting Information:

Contrast-enhanced optical coherence tomography with picomolar sensitivity for functional *in vivo* imaging

Orly Liba[a,b,d,e,1], Elliott D. SoRelle[a,b,c,d,1], Debasish Sen[a,b,d], and Adam de la Zerda[a,b,c,d,e,2]

[a]Molecular Imaging Program at Stanford, [b]Bio-X Program, and [c]Biophysics Program, [d]Departments of Structural Biology, and [e]Electrical Engineering, Stanford University, 299 Campus Drive, Stanford, California 94305

Author Contributions: O.L., E.D.S., D.S., and A.dlZ. designed research; O.L. and E.D.S. performed research, analyzed data, and wrote the paper.

[1]O.L. and E.D.S. contributed equally to this work
[2]To whom correspondence should be addressed.

Adam de la Zerda
Assistant Professor of Structural Biology
Stanford University
299 Campus Dr. West
Stanford, CA 94305
Tel: (650) 721-5469
Email adlz@stanford.edu

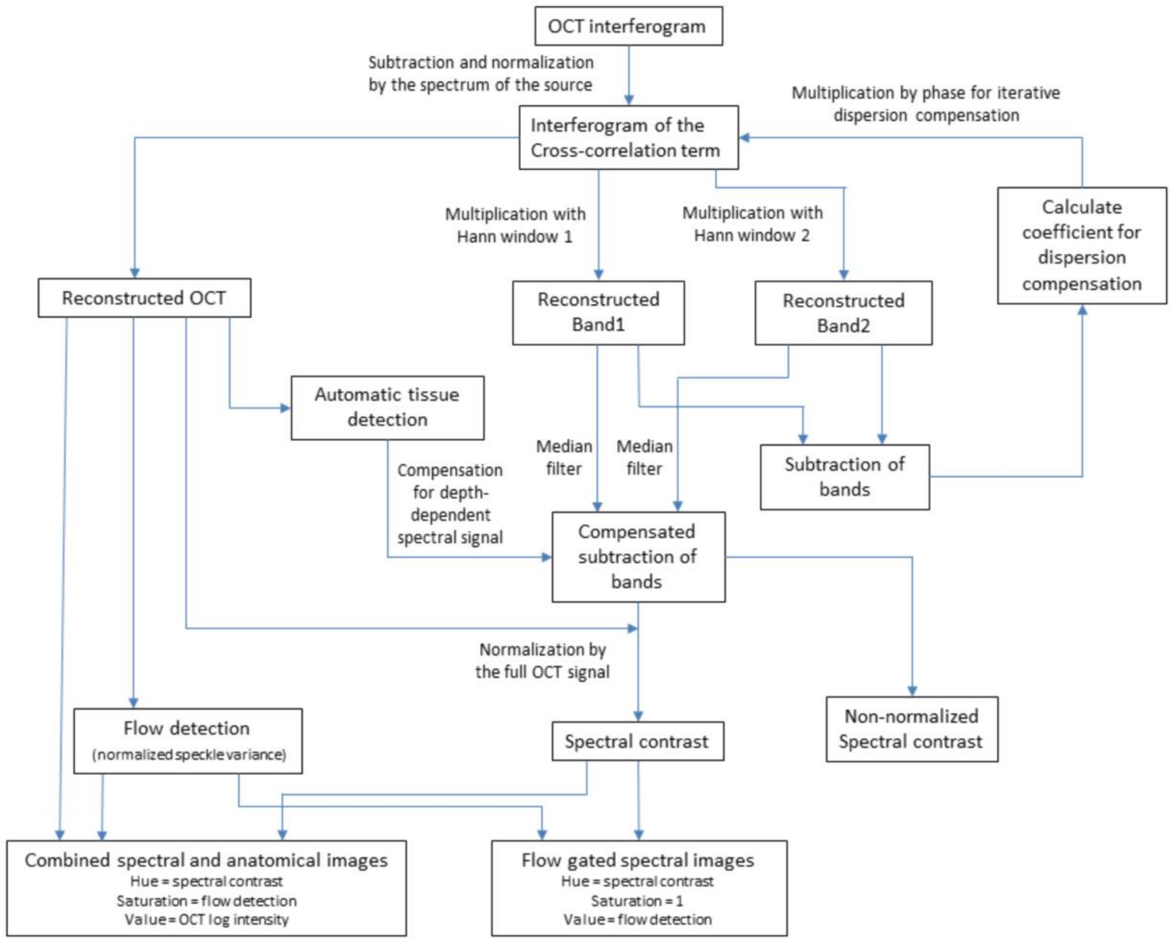

Figure S1. Block diagram of the post-processing steps from the raw data to the contrast-enhanced spectral images.

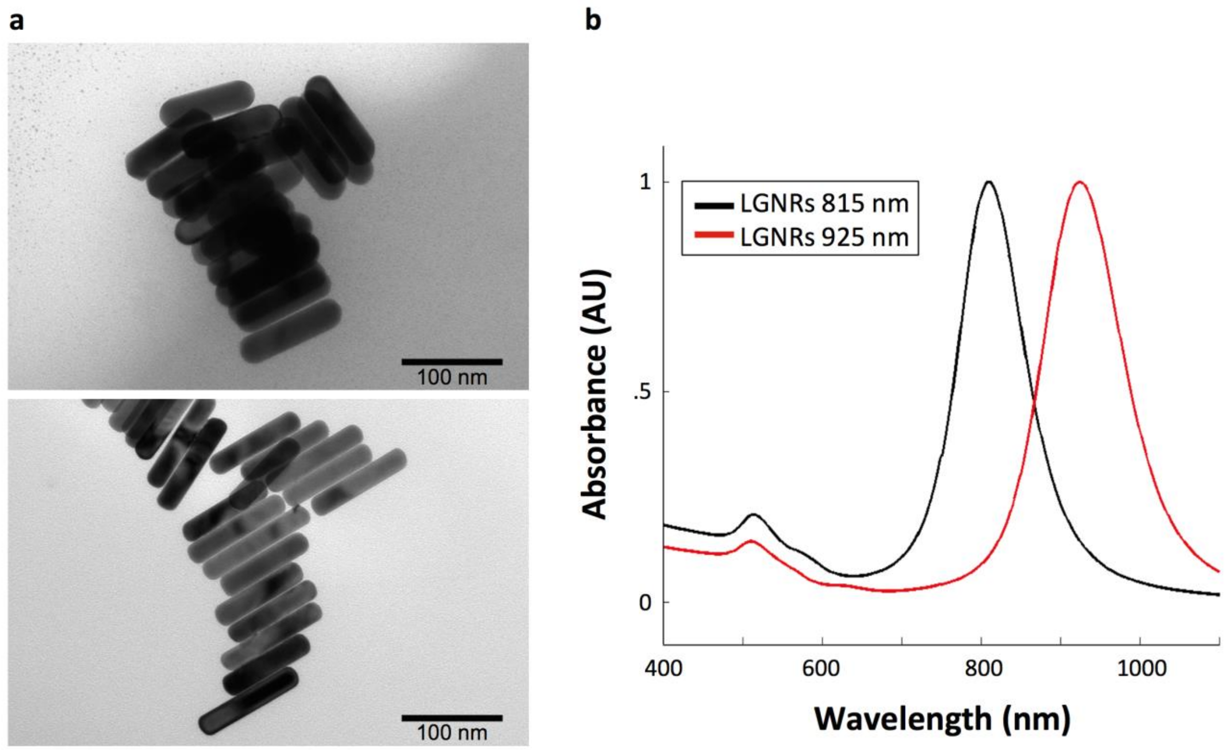

Figure S2. Physical characterization of LGNRs. (a) Representative TEM images of LGNRs. Top: LGNRs with average length of 107 ± 7 nm, average width of 32 ± 2 nm, and average aspect ratio of 3.4 (N=20). Bottom: LGNRs with average length of 105 ± 7 nm, average width of 22 ± 1 nm, and average aspect ratio of 4.8 (N=20). Scale bars are 100 nm. All values are presented as mean ± SD. (b) Vis-NIR absorbance spectra from LGNRs. LGNRs with an aspect ratio of 3.4 exhibit 815 nm peak longitudinal plasmonic resonance (black curve) and LGNRs with an aspect ratio of 4.8 exhibit 925 nm peak longitudinal plasmonic resonance (red curve). Both batches of LGNRs are highly monodisperse, as evidenced by their narrow spectral bandwidths and high ratios of longitudinal to transverse peak absorbance.

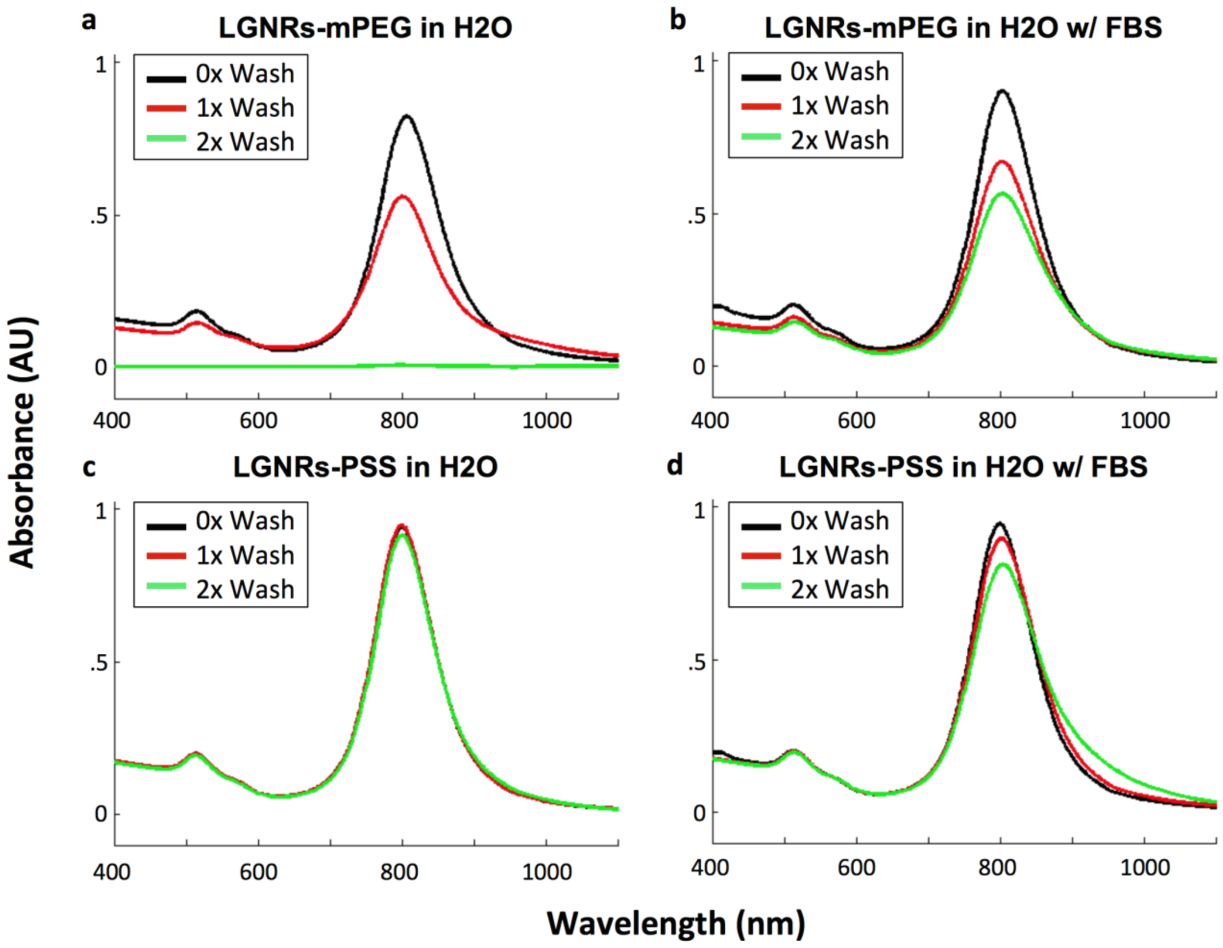

Figure S3. Evaluation of LGNR stability in aqueous media by spectral measurements. (a) LGNRs-mPEG do not remain stable through multiple washes in water. LGNRs-mPEG aggregate and crash out of solution, as evidenced by spectral peak broadening and reduction of peak absorbance after washing. (b) LGNRs-mPEG incubated with 10% FBS for 1 hour also exhibit instability, as indicated by reduced peak absorbance after incubation and washing. (c) LGNRs-PSS are highly stable in water through multiple rounds of washing by centrifugation and resuspension. Stability is evidenced by the fact that the absorbance properties of the particles do not change after each wash. (d) LGNRs-PSS incubated with 10% FBS for 1 hour remain largely stable through multiple washes. This result demonstrates that LGNRs-PSS are stable in the presence of proteins and biological media.

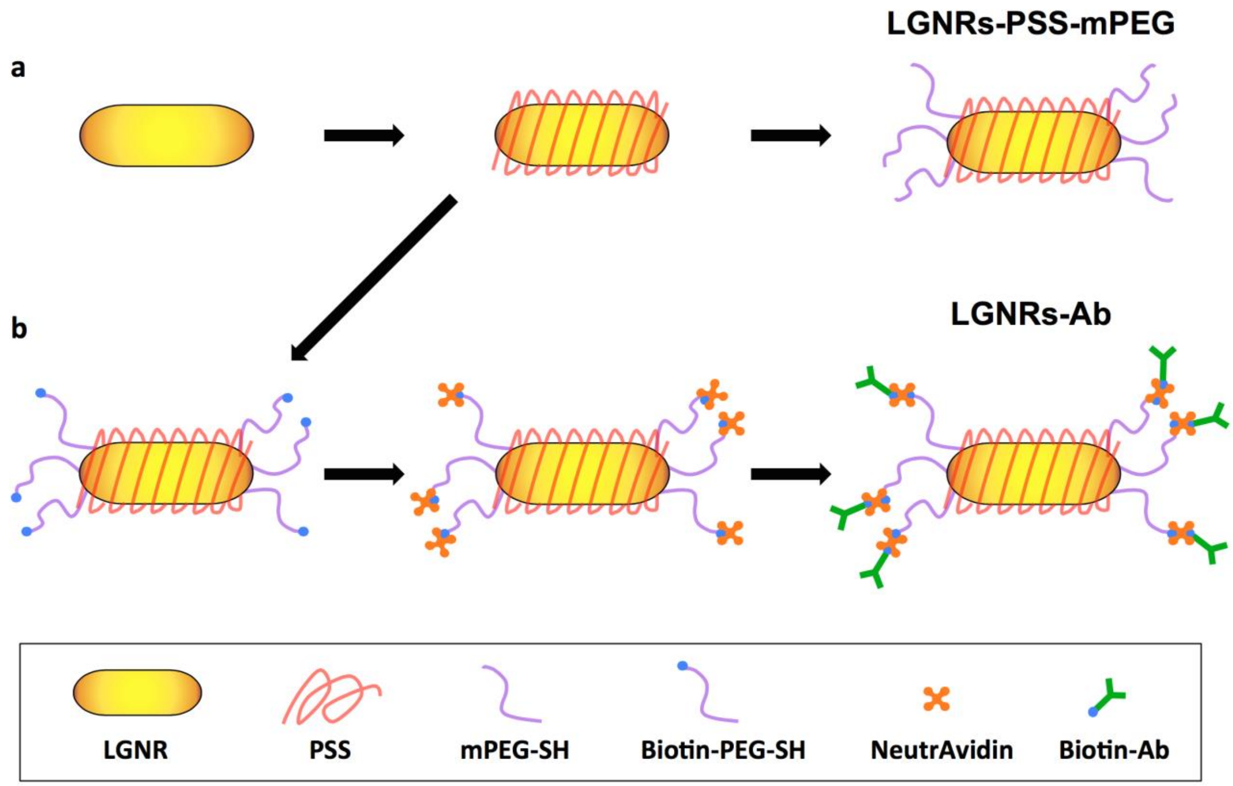

Figure S4. Surface coating strategies for LGNRs. (a) After synthesis, LGNRs are incubated with PSS and washed to produce LGNRs-PSS, which have negatively-charged surfaces. These particles are robustly stable in water, likely due to strong electrostatic repulsion among particles. LGNRs-PSS are subsequently reacted with mPEG-SH and washed to produce LGNRs-PSS-mPEG for use in lymph imaging experiments. (b) Alternatively, LGNRs-PSS are incubated with Biotin-PEG-SH and washed to produce LGNRs-PSS-PEG-Biotin. The free biotin groups bind with high affinity to NeutrAvidin upon incubation, producing LGNRs-PSS-PEG-Biotin-NA. After washing, these particles are incubated with biotinylated IgG1 antibodies and washed, yielding LGNRs-Ab. LGNRs-Ab were used in all intravenous injection experiments. The NeutrAvidin-Biotin interaction provides a platform for the conjugation of any biotinylated antibody of choice for future molecular targeting applications of LGNRs.

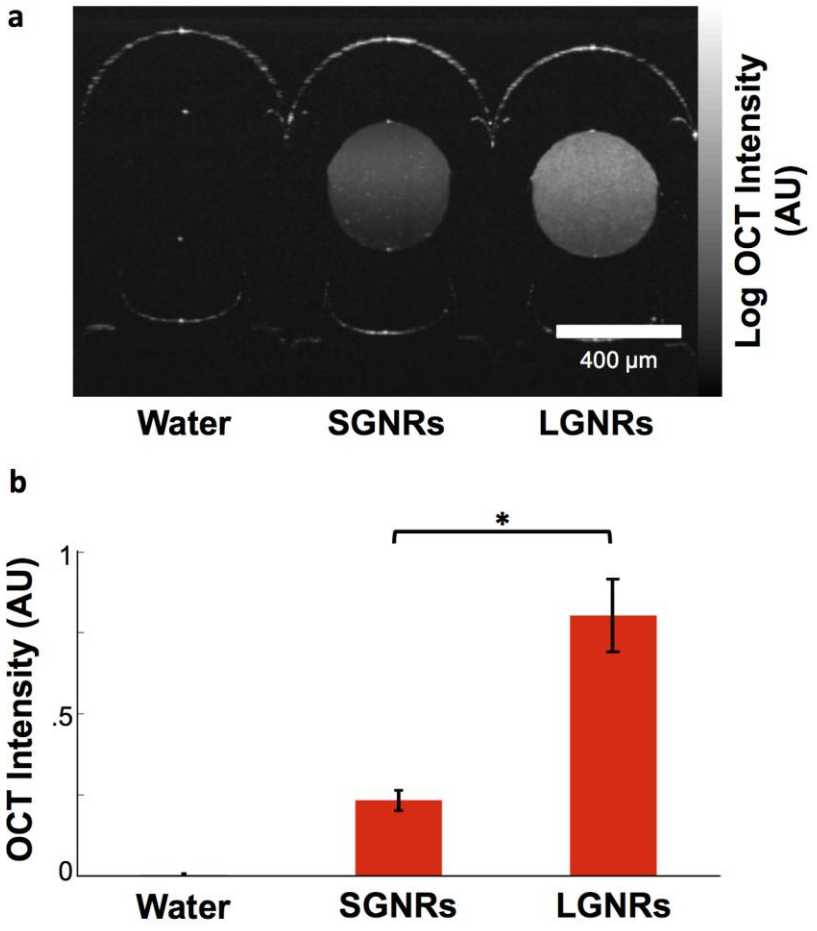

Figure S5. OCT signal comparison of SGNRs and LGNRs. (a) Log OCT intensity of water, SGNRs, and LGNRs in capillary tubes. The GNRs are at equal mg/mL and optical density (OD 1.25). At this OD, the concentration of LGNRs is 62.5 pM and the concentration of SGNRs is 500 pM. Even though the mass of gold in each GNR sample is equivalent, the LGNRs have a larger scattering- to-absorbance ratio and thus the OCT signal from LGNRs is greater. (b) OCT intensity (linear scale) of the samples in (a), collected around the focal plane. The OCT signal from LGNRs is ~3.5-fold stronger compared to the SGNRs at equivalent OD (*$p<0.001$). Quantitative data are presented as mean ± SD.

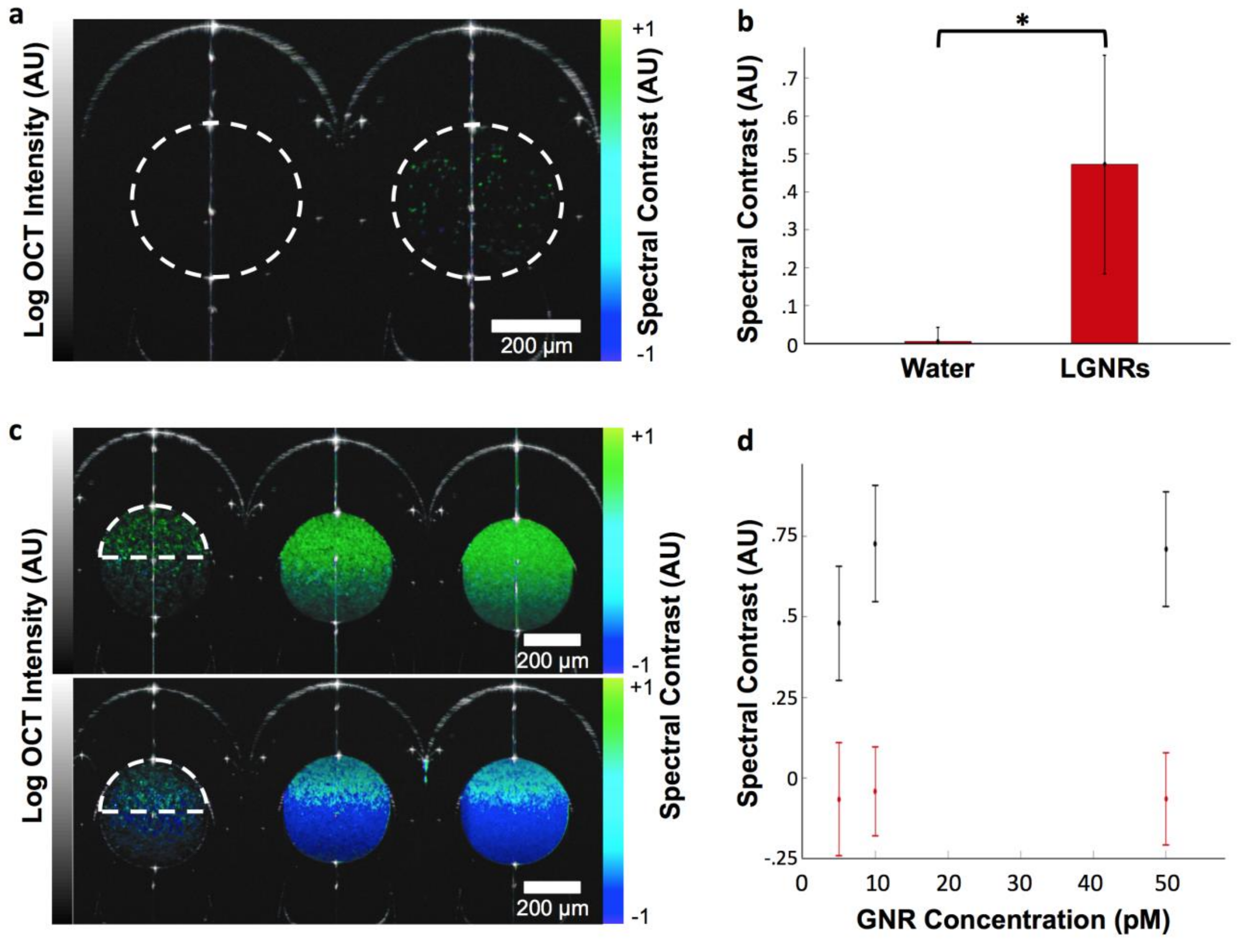

Figure S6. Detection of LGNRs in water. (a) Capillary tubes with water (left) and LGNRs with a peak at 815 nm (right) at a low concentration (500 fM). At this concentration, individual LGNRs can be identified by their spectral contrast. Multiplying this LGNR concentration by the imaged volume of the capillary tube (250 pL) and Avogadro's number predicts that ~75 LGNRs should be present in the image, which is consistent with the observed number of bright spectral puncta (~65). (b) Spectral contrast of water versus individual LGNRs (*$p < 0.001$). (c) Analysis of spectral contrast of LGNRs with a peak at 815 nm (top) and 925 nm (bottom) in water, at concentrations of 5, 10 and 50 pM. The dashed lines show the regions that were analyzed for quantification of spectral contrast. (d) Quantification of spectral contrast of 815 nm LGNRs (black) and 925 nm LGNRs (red) as a function of LGNR concentration. Each measurement is the average spectral contrast over 4 A-scans on the top half of the capillary tube, as shown in (c). Depth-dependent compensation was not applied for these images. Therefore, the top half of the capillary tube was selected for analysis since it is only minimally affected by absorption and chromatic aberrations. Scale bars are 200 μm. Quantitative data are presented as mean ± SD.

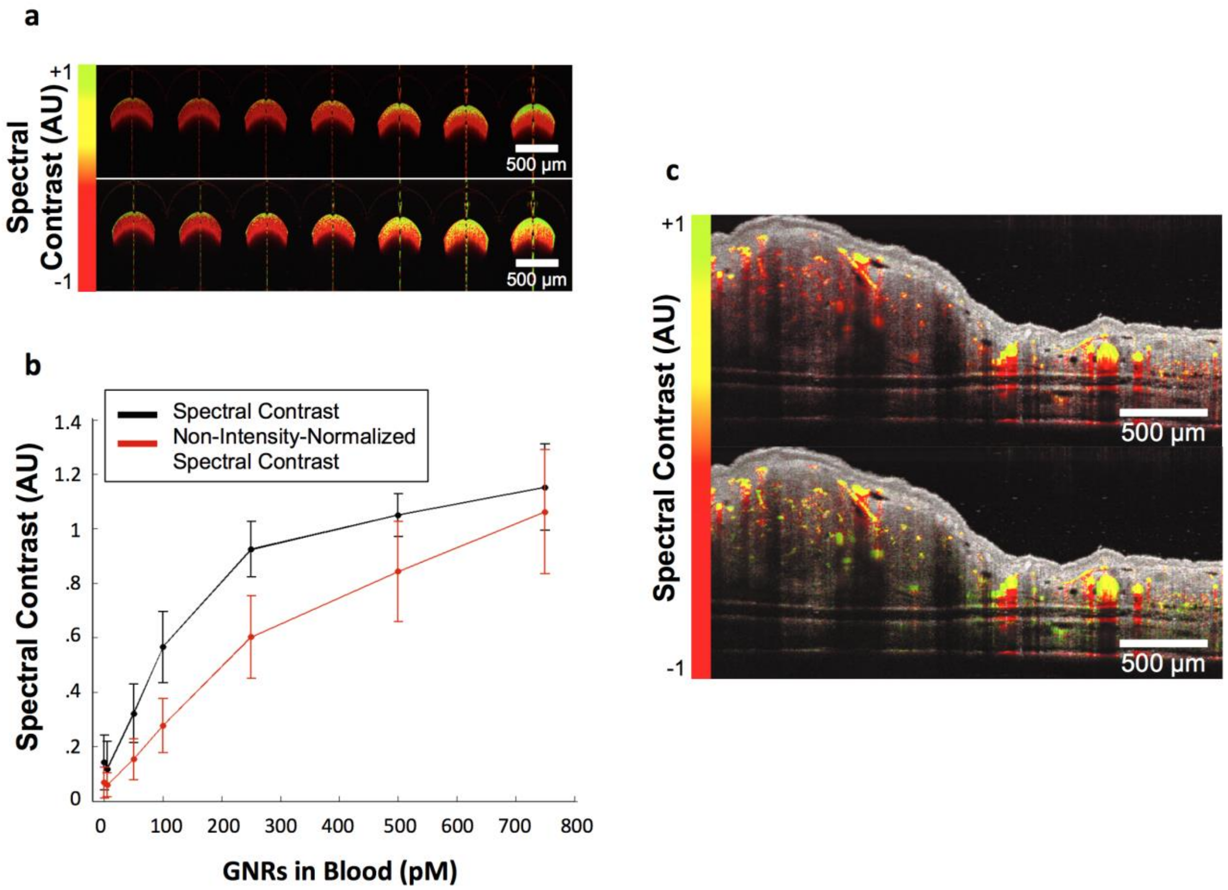

Figure S7. *In vitro* and *in vivo* evaluation of LGNR spectral contrast with and without normalization. (a) The non-normalized (top) and normalized spectral contrast (bottom) of 815 nm LGNRs in blood, without depth-dependent compensation, showing the change in hue from red to yellow-green as LGNR concentration increases. (b) Quantification of the non-normalized and normalized spectral contrast, showing a more linear behavior for the non-normalized signal as a function of LGNR concentration. Thus, the non-normalized spectral contrast could be used for relative quantification of the LGNRs in blood. Each measurement is the average spectral contrast over 4 A-scans on the top of the capillary tube to reduce the effect of absorption. Quantitative data are presented as mean ± SD. (c) Non-normalized (top) and normalized (bottom) spectrally enhanced OCT images of a tumor on the pinna after IV injection of 815 nm LGNRs-Ab. The blood vessels deep inside the tumor are red and not yellow-green in the non-normalized image because the non-normalized spectral contrast decreases inside the tumor due to OCT signal decrease caused by absorption of the tissue. Scale bars are 500 µm.

a

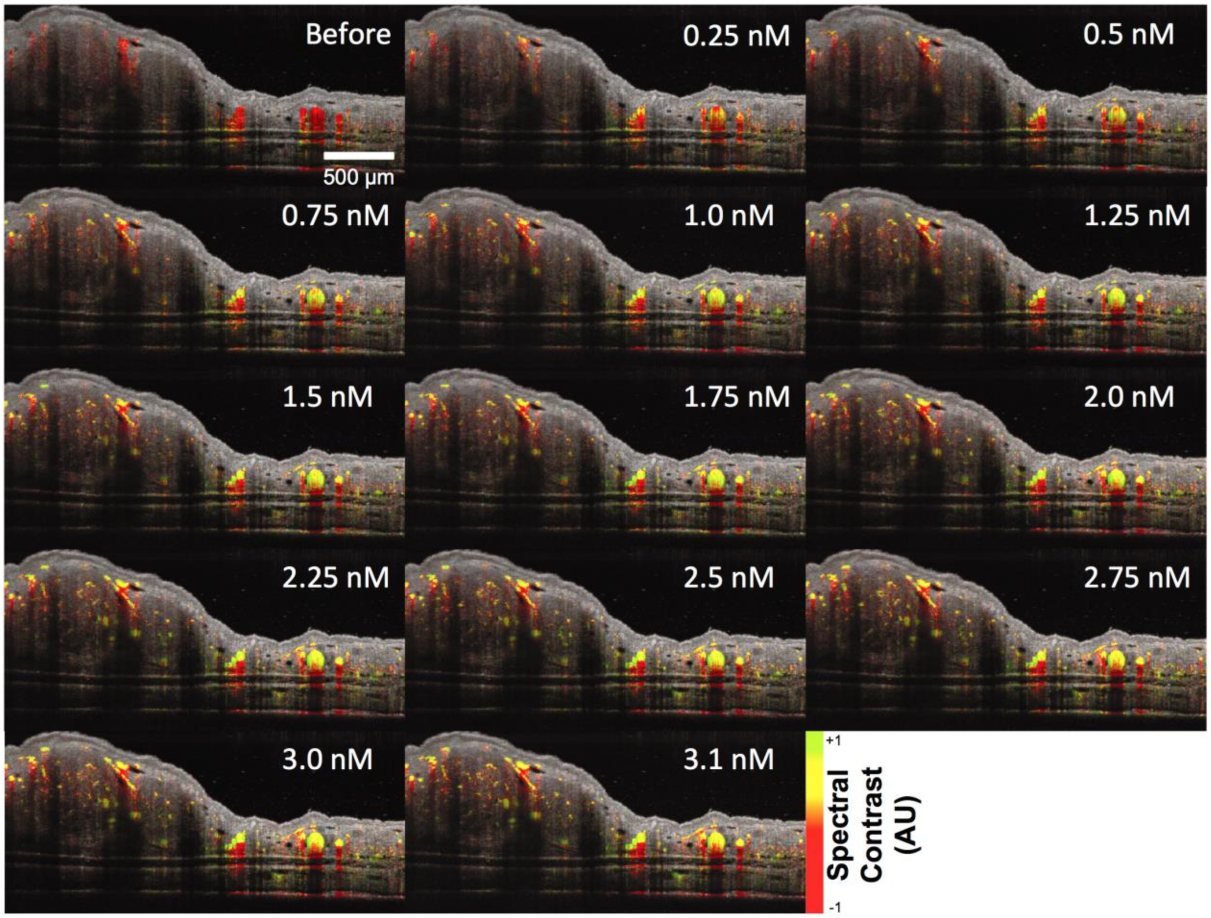
Before
500 µm
0.25 nM
0.5 nM
0.75 nM
1.0 nM
1.25 nM
1.5 nM
1.75 nM
2.0 nM
2.25 nM
2.5 nM
2.75 nM
3.0 nM
3.1 nM
+1
Spectral Contrast (AU)
-1

b

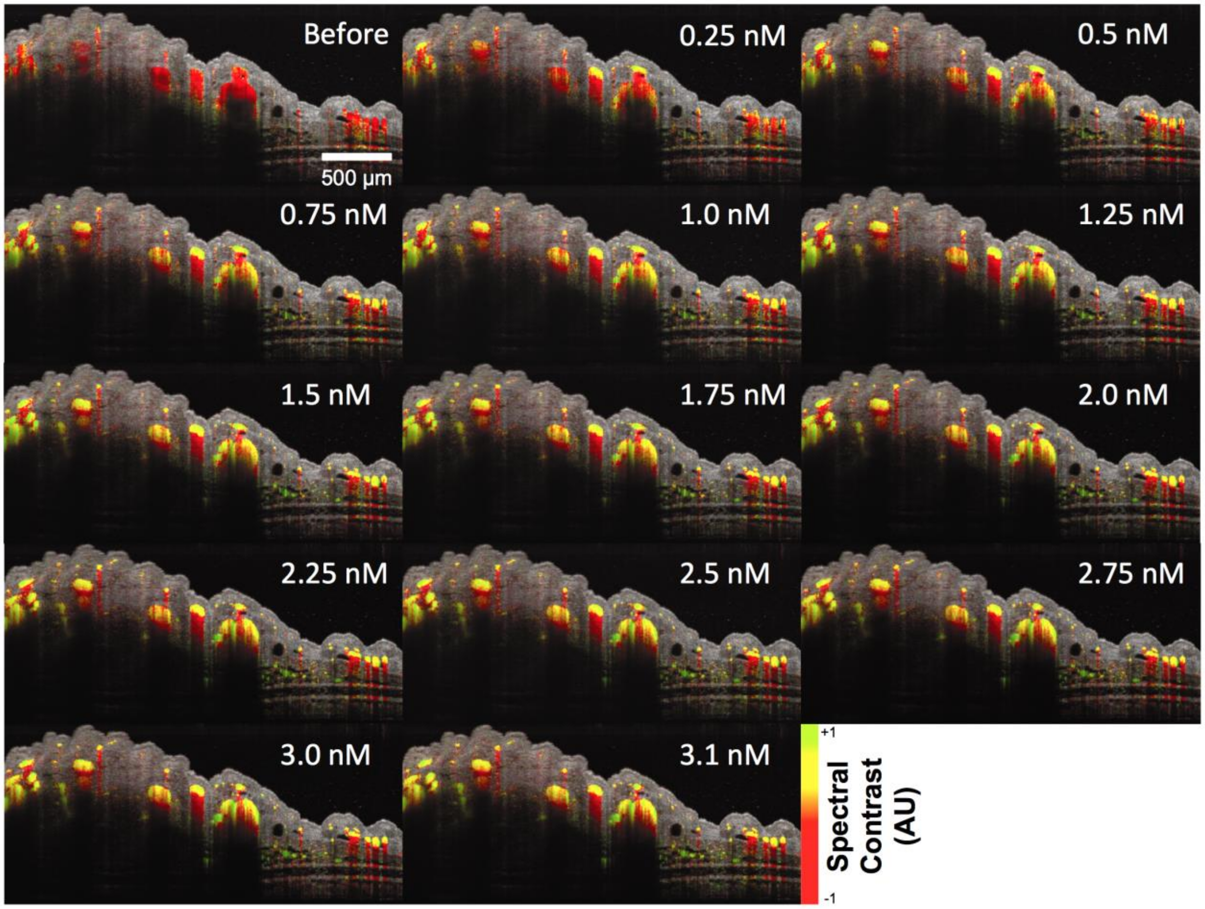
Before
500 μm
0.25 nM
0.5 nM
0.75 nM
1.0 nM
1.25 nM
1.5 nM
1.75 nM
2.0 nM
2.25 nM
2.5 nM
2.75 nM
3.0 nM
3.1 nM
+1
Spectral Contrast (AU)
-1

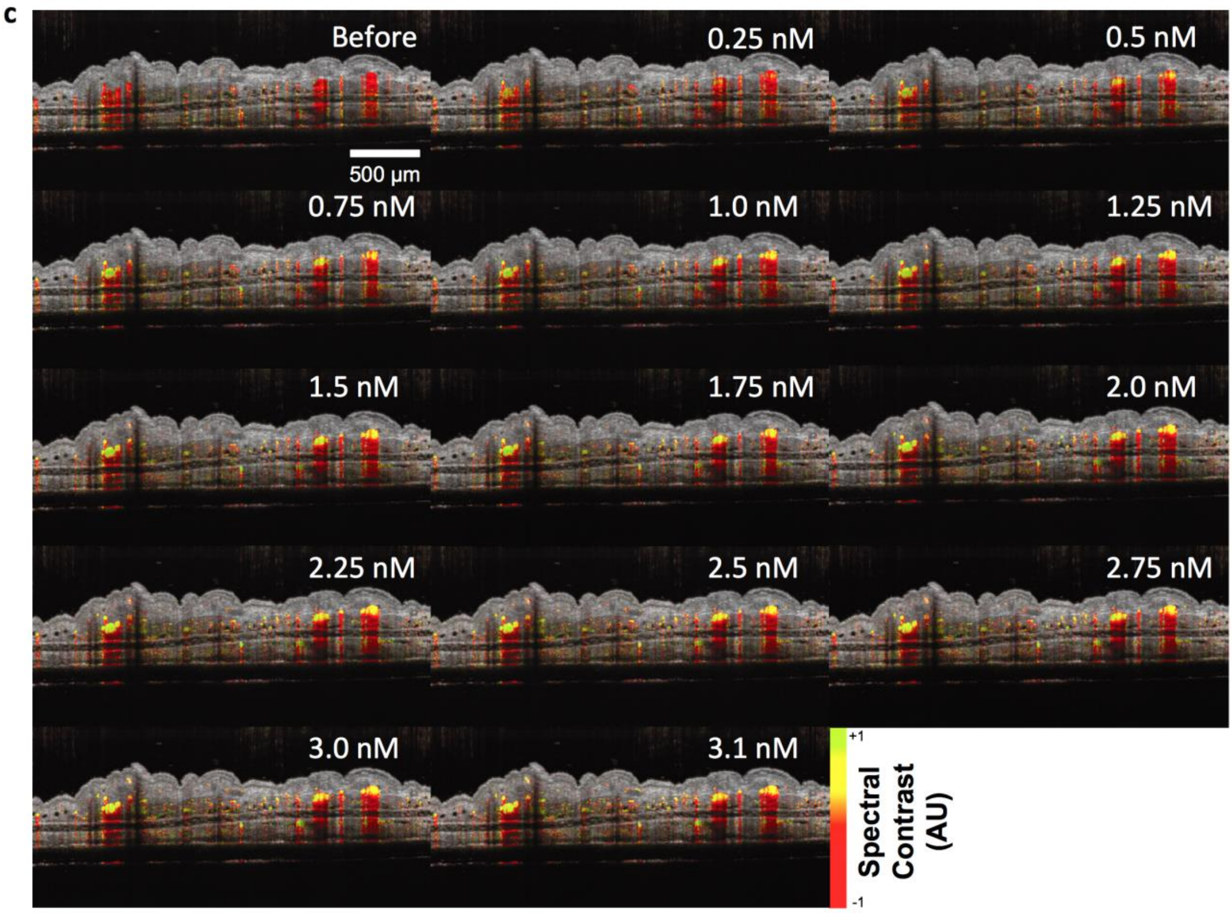

Figure S8. Contrast-enhanced B-scans of three mice during incremental IV injections of 815 nm LGNRs-Ab. (a) and (b) show scans across a tumor and the healthy tissue adjacent to it. (c) shows a scan of an ear without a tumor. Concentrations are estimated from the concentration of injected LGNRs-Ab and the volume of blood in a healthy 25g mouse (~2 mL). Images are composed of anatomical information in gray scale and flow-gated spectral contrast in color. Scale bars are 500 μm.

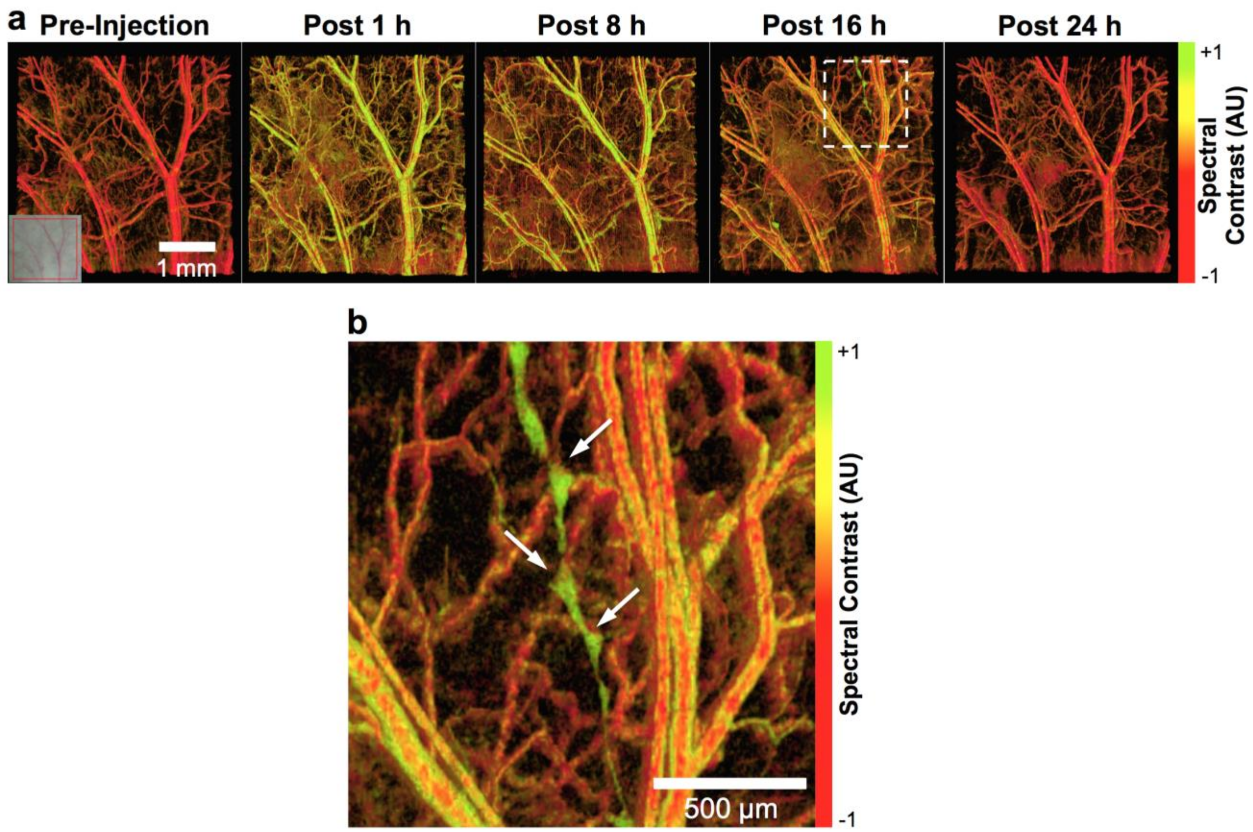

Figure S9. Additional images from an IV injection experiment with 815 nm LGNRs-Ab. (a) Images of healthy mouse ear acquired before and after (t=0, 8, 16, and 24 h) injection. Spectral signal from LGNRs-Ab is clear immediately following IV injection and gradually clears from circulation over 24 h. Scale bar is 1 mm. (b) Zoom of 16 h image (white dashed square) from (a) showing LGNR-Ab presence in lymph vessel. Despite IV injection, some LGNRs-Ab extravasated from blood vessels into ear tissue during the imaging time course. These LGNRs-Ab were collected and drained by lymphatic vessels within the ear pinna. White arrows indicate the positions of valves at the ends of individual lymphangions. Scale bar is 500 µm.

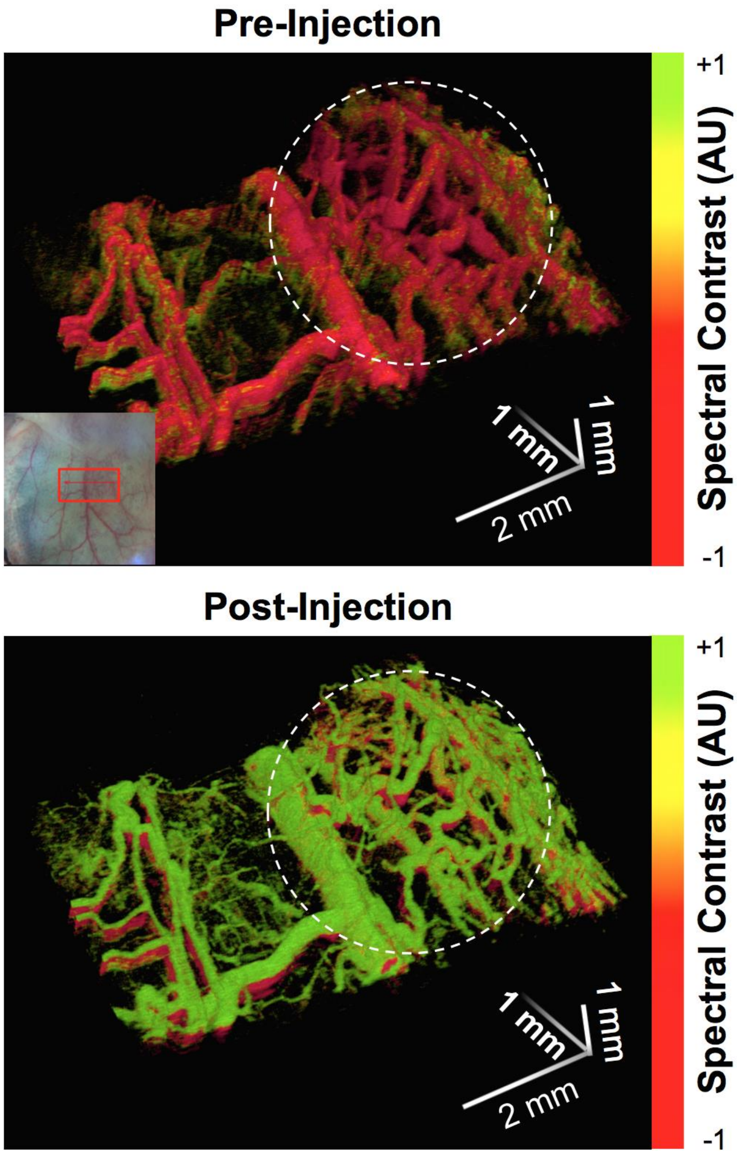

Figure S10. Additional images from an IV injection experiment with 815 nm LGNRs-Ab. Pre-injection and post-injection (t=0 h) images acquired from a separate tumor-bearing mouse IV injected with LGNRs-Ab. The tumor tissue region (white dashed circle) displays more tortuous vasculature than nearby healthy tissue. Additional small blood vessels can be seen after LGNR injection. Imaged fields of view are 4 mm x 2 mm x 1 mm.

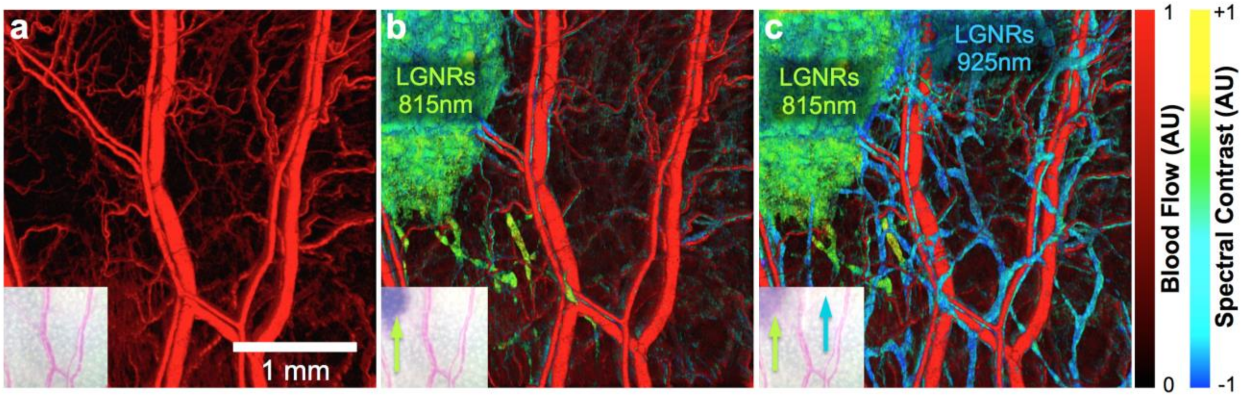

Figure S11. A third example of LGNRs-PSS-mPEG draining into the lymph vessels of the pinna *in vivo*. (a) *En face* view of flow detection, showing blood vessels (in red) prior to LGNR-PSS-mPEG injection. The inset is a photograph of the ear showing the scanned region. (b) Spectral contrast showing the first injection of 815 nm LGNRs (shown as green) with an overlay of the blood vessels from (a). (c) As in (b), after the second injection, of 925 nm LGNRs (shown as cyan-blue). The LGNRs are filling the lymph vessels and draining from the injection site, at the edge of the ear (top of image) to the base of the ear (bottom of image), revealing a vast network of lymph vessels in (c).
